# Psychometric functions from multiple responses: Dedicated to the memory of Colin L. Mallows

**DOI:** 10.3758/s13428-025-02828-7

**Published:** 2026-06-29

**Authors:** Saul Sternberg, Ronald L. Knoll, Colin L. Mallows

**Affiliations:** 1 University of Pennsylvania, Philadelphia, PA USA; 2 Retired, Austin, TX USA; 3 Flemington, NJ USA

**Keywords:** Psychophysics, Psychometric function, Perception, Perceptual-decision models, Temporal-order judgment

## Abstract

By using three or more ordered response categories and varying the stimulus feature being judged over a large range, it is possible to generate a family of psychometric functions (PMFs), each based on a different partition of the responses. An earlier paper showed how, when it is treated as a probability distribution, the traditional single PMF based on binary-choice data can be decomposed into sensory and decision components, expressed as two independent random variables that are summed to create the PMF. Here we extend this development to the multiple-response procedure, and use it to elucidate the relations among the spreads and shapes of the resulting family of PMFs, which can be described by their first four cumulants. For example, we determine conditions under which the PMFs can have the same spread and shape, differing only by translation on the stimulus axis. Whereas PMFs depend on both sensory and decision processes, differences among the PMFs in a family depend only on the decision processes. Application of this *multiple-PMF method* to several decision models, whose evaluations depend on the PMF cumulants, shows it to have greater power than the single-PMF method for understanding the perceptual process. Although this work was inspired by experiments on the perception of temporal order, it can be applied to experiments where features of stimuli other than their occurrence times are being compared, such as the pitch of tones or the brightness of lights.

## 1 Introduction

Often, in experiments where confidence ratings are used to measure discriminability or test psychophysical models, relatively few distinct values of the stimulus variable are presented – sometimes only two – yielding data that cannot provide information about the shape of the psychometric function (PMF).^1^ On the other hand, when the PMF is of primary interest, a larger range of stimulus values is presented, but the observer is often required to choose between only two response alternatives. Suppose that in the same experiment in which the stimulus or stimulus difference varies from trial to trial over a large range, the observer is required to choose the response from an ordered set of *n* > 2 categories: Examples of such categories include binary decisions with confidence ratings; “higher”, “same”, or “lower” in comparing two pitches; and “first”, “simultaneous”, or “second” in judging temporal order. Then each of the *n* − 1 partitions of the set of response categories can be used to generate a distinct PMF.^2^ The potential usefulness of some of the relations among the members of such a family of PMFs for answering theoretical questions has occasionally been recognized. For examples, see Nachmias & Steinman (1963) and Eijkman, Thijssen, & Vendrik (1966) in vision, Thijssen & Vendrik (1968) in audition, Ulrich (1987) in temporal-order perception, and Rammsayer & Ulrich (2001) in duration discrimination.

In this paper, we first review the representation of the PMF derived from binary-response temporal-order judgments in terms of the components of a general independent-channels model discussed previously by Sternberg and Knoll (1973), and then generalize this treatment to the multiple-response procedure and other perceptual domains. We find the conditions under which the family of PMFs generated from the procedure can be expected all to have the same spread and shape – i.e., to be parallel, differing only by translation on the stimulus axis. We also provide examples to show how relations among the shapes of the family of functions can provide tests of models that a single function would not permit and, in general, can help to decompose the observed data into separate contributions from sensory and decision processes.

One reason for our interest in these issues was the findings by L. G. Allan (1975a) of systematically non-parallel sets of PMFs from a procedure in which the observer was required on each trial to rate the offsets of a light and a tone as simultaneous or successive, and then to judge their order as light first or tone first, thereby generating four ordered response categories.^3^ In another study (Allan, 1975b), the observer was required on each trial to judge the order of two offsets, and rate her confidence in this judgment as high (“certain”) or low (“uncertain”), again generating four ordered response categories. Data from four response categories (**R =** 1, 2, 3, 4) can be used to generate a family of three PMFs: *F*_1_ **=** *Pr*{**R** > 1}, *F*_2_ **=** *Pr*{**R** > 2}, and *F*_3_ **=** *Pr*{**R** > 3}; in both studies Allan found that the middle function (*F*_2_) was relatively symmetric while *F*_1_ was positively skewed and *F*_3_ negatively skewed (see Fig. 2). There was also a tendency in Allan’s data for the variance of *F*_2_ to be greater than that of *F*_1_ or *F*_3_.

What, if anything, might justify or explain our intuition that the members of such PMF families should be parallel? And, if they are not parallel, what can we learn from the relations among their locations, spreads, and shapes? 

Consider the following experiment on temporal-order perception: The stimuli are *X* presented at time *t*_*x*_ and *Y* presented at time *t*_*y*_. From trial to trial, the time difference *t*_*y*_ − *t*_*x*_ **=** Δ*t* takes on various values that can be positive, zero, or negative. After each presentation, the observer judges whether *X* appeared to occur before *Y* (response ″*t*_*x*_ < *t*_*y*_″) or after *Y* (response ″*t*_*x*_ > *t*_*y*_″), and also provides a rating of confidence in the judgment. If we ignore the confidence ratings, the data can be used to estimate a traditional PMF,

1 $$F\left(\Delta t\right)=Pr\{^{\prime\prime} {t}_{x}<{t}_{y}^{\prime\prime} \left|\Delta t\right.\},$$

in which the probability of the judgment that *X* preceded *Y* typically increases, often monotonically, with the stimulus variable Δ*t* over a range from zero to one and when the function that represents the decision process may also be nonmonotonic. Just as the shape of a distribution can be characterized by its cumulants,^4^ so can the shape of a PMF, even when the PMF is nonmonotonic. (For examples of nonmonotonic PMFs for temporal-order judgments, see Figure 2 and Kelber & Ulrich, 2024, and for nonmonotonic PMFs from simulation of sensory judgment models, see Appendix C.) In describing these computations, we shall use the language of probability, recognizing that some concepts may not be conventional.

## 2 Models for the psychometric function generated by binary choice data

In an earlier paper, Sternberg and Knoll (1973) showed how *F*(Δ*t*) could be described in terms of the components of a general independent-channels model. (We refer the reader to that paper for details.) In this model, which is a generalization of numerous models that have been proposed for temporal-order judgments, a “decision function” converts a difference in central “arrival times” of two sensory signals into an order judgment. Let the arrival times^5^ of stimuli *X* and *Y* be represented by the random variables **A**_*x*_ and **A**_*y*_, respectively. The sensory value **S**, here the *arrival-time difference* **A**_*y*_ − **A**_*x*_ depends, in turn, on the difference *d* **=** *t*_*y*_ − *t*_*x*_ between stimulation times *t *_*x*_ and *t*_*y*_ and separate *arrival latencies* **L**_*x*_ and **L**_*y*_ according to

2 $$\mathbf{S}={\mathbf{A}}_{y}-{\mathbf{A}}_{x}=\left({t}_{y}+{\mathbf{L}}_{y}\right)-\left({t}_{x}+{\mathbf{L}}_{x}\right)={\mathbf{L}}_{y}-{\mathbf{L}}_{x}+d.$$

The decision rule induces a decision function *D* on values of the sensory variable **S = A**_*y*_ − **A**_*x*_, associating a decision probability with each value of **S**, such that for any value of *S*,

3 $$D\left(S\right)=Pr\left\{"{t}_{x}<{t}_{y}" |\mathbf{S}=S\right\}.$$

A simple decision rule, and one that is often assumed, is the deterministic decision rule: the observer reports *X* before *Y* (or *X* lower in pitch or luminance than *Y*) if and only if the difference is non-negative (i.e., matches or exceeds a criterion of zero). Thus, *D*(*S*) **=** 0 when *S* < 0, and *D*(*S*) **=** 1, otherwise. This rule is readily generalized to an arbitrary criterion, *θ*:

4 $$D\left(S\right)=\left\{\begin{array}{c}0,S<\theta \\ 1,S\ge \theta .\end{array}\right.$$

Much can be gained by treating the PMF, *F*(*δ*) (which is sometimes assumed to be a strictly monotonic increasing function) as the distribution function of a random variable **F**:

5 $$F\left(\delta \right)=Pr\left\{\mathbf{F}\le \delta \right\}.$$

Sternberg and Knoll (1973, Section II) showed that for the decision rule expressed by Eq. (4),

6 $$\mathbf{F}={\mathbf{A}}_{y}-{\mathbf{A}}_{x}+\theta =\mathbf{S}+\theta .$$

They also showed that the decision function D need not be a step function; as long as it is a nondecreasing function, it can be regarded as the distribution function of a random variable **D** that is stochastically independent of **A**_*y*_ − **A**_*x*_, and Eq. (6) can be generalized^6^ as

7 $$\mathbf{F}=\mathbf{S}+\mathbf{D}.$$

The PMF can thus be expressed additively in terms of the sensory variable **S** (**= A**_*y*_ − **A**_*x*_, for temporal-order judgments) and the decision process (**D**). That is, thinking of the PMF as the distribution function of a random variable (**F**), it can be expressed as the convolution of the distribution of the sensory feature being judged (such as the arrival-time difference, or the pitch difference), with a stochastically independent distribution that represents the decision process. This is equivalent to thinking of the PMF as the distribution of a random variable (**F**) that is the sum of two stochastically independent random variables, one (**S**) whose distribution is that of the sensory variable, and the other (**D**) whose distribution corresponds to the decision process. Given this representation, it follows that the cumulants of the PMF can be written as sums of the corresponding cumulants of **S** and **D**: *κ*_*m*_(**F**) **=** *κ*_*m*_(**S**) + *κ*_*m*_(**D**). This *cumulant-additivity* property means, for example, that the cumulants of the sensory variable (e.g., the arrival-time difference, or the pitch difference) can be estimated by subtracting the corresponding cumulants of the (assumed) decision random variable, **D**, from those of the PMF.^7^ And it also means that differences between cumulants of PMFs based on different partitions of the responses are independent of the sensory variable: One implication is expressed by Eq. (11), below.

Many plausible decision mechanisms generate functions D that are not step functions.^8^ One possibility, for example, is a rule like the deterministic one (Eq. 4) but with a criterion *θ* that fluctuates from trial to trial. Let **B** represent the fluctuating criterion. Then, since *D*(*S*) ≡ *Pr*{″*t*_*x*_ < *t*_*y*_″ | **S =** *S*} **=** *Pr*{**B ≤** *S*}, the decision function D can be identified with the (cumulative) distribution of criterion values across trials. Thus D must be a nondecreasing function, **D** can be identified with **B**, and, as described by Eq. (7), **F** is the convolution of the distribution of sensory values with this criterion distribution.

## 3 Families of psychometric functions from multiple responses

The arguments outlined above can be generalized to the *family* of PMFs generated by partitioning an ordered set of responses at different levels. Suppose the observer uses responses **R =** 1, 2, ..., *n*. One possibility, e.g., is that **R =** 1 represents high confidence that *X* followed *Y* or that *X* had a higher pitch than *Y* (typically associated with large negative **S** values), and that **R =** *n* represents high confidence that *X* preceded *Y* or that *X* had a lower pitch than *Y* (typically associated with large positive **S** values). Then the PMF of Eq. (1) can be replaced by a family of *n* − 1 such functions, *F*_*i*_(*δ*), *i* **=** 1, 2, ..., *n* − 1, with

8 $${F}_{i}\left(\delta \right)= Pr\left\{\mathbf{R}>i | \delta \right\}.$$

The function *F*_*i*_(*δ*) results from partitioning the responses into 0 < **R ≤** *i* and *i* < **R ≤** *n*, *i* **=** 1, 2, ..., *n* − 1. Again, *F*_*i*_(*δ*) can be regarded as the distribution of a random variable **F**_*i*_. That is, by analogy with Eq. (5),

9 $${F}_{i}\left(\delta \right)=Pr\left\{{\mathbf{F}}_{i}\le \delta \right\}.$$

Given the multiple-response procedure, where the *i*^th^ partition of the responses is associated with a distinct decision process, represented by **D**_*i*_, and a distinct PMF, *F*_*i*_, Eq. (7) becomes

10 $${\mathbf F}_i=\mathbf S+{\mathbf{D}}_i\text.$$

Let *κ*_*m*_(**D**_*i*_) be the *m*^th^ cumulant of **D**_*i*_. Because **S** is invariant across different members {**F**_*i*_} of a PMF family, together with cumulant additivity for stochastically independent random variables, cumulant differences *κ*_*m*_(**D**_*i*_) − *κ*_*m*_(**D**_*j*_) among the decision processes will produce equal cumulant differences *κ*_*m*_(**F**_*i*_) − *κ*_*m*_(**F**_*j*_) among the PMFs, and thus be observable, and for any pair of PMFs, **F**_*i*_ and **F**_*j*_, in the same family,

11 $$\left[\kappa_m({\mathbf F}_i)-\kappa_m({\mathbf D}_{i})\rbrack-\lbrack\kappa_m({\mathbf F}_{j})-\kappa_m({\mathbf D}_j)\right]=0,\;m=1,2,....$$

Eq. (11) provides tests of the hypothesized decision process.^9^

Because, for all *δ*, *Pr*{**R** > *i*} ≥ *Pr*{**R** > *i* + 1}, it follows from the definition in Eq. (8) that the *F*_*i*_ are characterized by a dominance property:

12 $$\begin{array}{cc}F_i\left(\delta\right)\geq F_{i+1}\left(\delta\right),-\infty<\delta<\infty,&i=1,2,...,n-2.\end{array}$$

That is, the larger the response index *i* the lower, and further to the right on the *δ*-axis the PMF lies. To elaborate further on the relationships among the PMFs requires a model.

## 4 Model creation and development

In each of Sections 5, 7, 8, and 9, we develop a model for the decision process that starts with values of the sensory variable, **S**, and produces multiple psychometric functions. Our purpose is to show some of the possibilities for using the multiple-pmf method to create and test models of the decision process. The model is initially specified (in Tables 5.1, 7.1, 8.1, and 9.1) by dividing the sensory variable, **S**, into a set of non-overlapping intervals that cover all possible values of **S**. Each interval is associated with either multiple responses with probabilities that sum to 1.0, or a single response. On each trial, the response is determined by the interval into which the value of **S** falls, together with the probabilities of the alternative responses associated with that interval. Thus, each model starts as a mapping, sometimes probabilistic, of the sensory variable onto responses. Each row of these tables corresponds to one of the alternative responses. The responses are ordered, so that when, for example, there are four responses (**R** = 1, 2, 3, 4), we can consider quantities such as *Pr*{**R** > 2}.

**Table 5.1.** Multiple-Response Deterministic Model: Response Probabilities in *n* intervals along the sensory **S = A**_*y*_ − **A**_*x*_ axis

**S** Interval	[−∞, *θ*_1_)	[*θ*_1_, *θ*_2_)	[*θ*_2_, *θ*_3_)	...	[*θ*_*n-*2_, *θ*_*n-*1_)	[*θ*_*n-*1_, +∞)
Response	**R** = 1	**R** = 2	**R** = 3	...	**R** = *n* − 1	**R** = *n*
*h*_1_ **=** *Pr*{**R =** 1}	1	0	0	...	0	0
*h*_2_ **=** *Pr*{**R =** 2}	0	1	0	...	0	0
*h*_3_ **=** *Pr*{**R =** 3}	0	0	1	...	0	0
...	...	...	...	...	...	...
*h*_*n*−1_ **=** *Pr*{**R =** *n* − 1}	0	0	0	...	1	0
*h*_*n*_ **=** *Pr*{**R =** *n*}	0	0	0	...	0	1

In Tables 5.2, 7.2, 8.2, and 9.2, the probabilities of individual responses (such as *Pr*{**R =** 2}) are converted into cumulative probabilities (such as *Pr*{**R** > 2}), thus creating the decision variables (such as **D**_2_). Each row of these tables describes the decision function for a different decision variable, which will correspond to a different psychometric function. In each of these models, the distribution of **D**_*i*_ has probabilities at only a small number of discrete values of the sensory variable.

**Table 5.2.** Multiple-Response Deterministic Model: Cumulative Distribution Functions of the {*D*_*i*_}

**S** Interval	[−∞, *θ*_1_)	[*θ*_1_, *θ*_2_)	[*θ*_2_, *θ*_3_)	...	[*θ*_*n−*__2_, *θ*_*n−*__1_)	[*θ*_*n−*__1_, +∞)
*D*_1_ **=** *Pr*{**R** > 1}	0	1	1	...	1	1
*D*_2_ **=** *Pr*{**R** > 2}	0	0	1	...	1	1
*D*_3_ **=** *Pr*{**R** > 3}	0	0	0	...	1	1
...	...	...	...	...	...	...
*D*_*n*_−_1_ **=** *Pr*{**R** > *n* − 1}	0	0	0	...	0	1

In Tables 5.3, 7.3, 8.3, and 9.3, the probability functions {*g*_*i*_} of the {**D**_*i*_} are provided, determined from values in the corresponding previous table. These values are used to calculate the cumulants of each of the {**D**_*i*_}, which are provided in Tables 5.4, 7.4, 8.4, and 9.4. An example of the calculation of cumulants is provided in Section 7.

**Table 5.3.** Multiple-Response Deterministic Model: Probability Functions of the {*D*_*i*_}

Function	Value				
	*θ*_1_	*θ*_2_	*θ*_3_	...	*θ*_n−1_
*g*_1_	1	0	0	...	0
*g*_2_	0	1	0	...	0
*g*_3_	0	0	1	...	0
...	...	...	...	...	...
g_*n*−1_	0	0	0	...	1

**Table 5.4.** Multiple-Response Deterministic Model: *D*_*i*_ Cumulants

	Distribution				
	*D*_1_	*D*_2_	*D*_3_	...	^*D*^*n*−1
*κ*_1_	*θ*_1_	*θ*_2_	*θ*_3_	...	*θ*_*n*−1_
*κ*_2_	0	0	0	...	0
*κ*_3_	0	0	0	...	0
*κ*_4_	0	0	0	...	0

Included in the assumptions that underlie the development of these models is the idea that the PMF can be thought of as a distribution function,^10^ and can thus be described by its cumulants. **F** can be expressed as the sum of two stochastically independent random variables: **S**, representing the sensory information, and **D**, representing the decision process (Sternberg & Knoll, 1973). In other words, the *PMF* is the convolution of the distribution of **S** with the distribution of **D**. It follows that the cumulants of the *PMF* are sums of the cumulants of **S** and the corresponding cumulants of **D**:

13 $$\begin{array}{cc}\kappa_m\left(\mathbf F\right)=\kappa_m\left(\mathbf S\right)+\kappa_m\left(\mathbf D\right),&m=\text{1, 2},\dots\end{array}$$

Suppose that a subject has to choose among *n* **=**3 alternative ordered responses, as in comparing the brightness of X and Y, by selecting one of the following responses:

- “X dimmer than Y” (**R** = 1),
- “X and Y equally bright” (**R** = 2), and
- “X brighter than Y” (**R** = 3).

Then the *n* − 1 **=** 2 partitions of the responses can each define a *PMF*:

$$\begin{array}{c}{F}_{1}= Pr(\mathbf{R}>1),\\ {F}_{2}= Pr(\mathbf{R}>2),\end{array}$$

(each a function of the luminance difference between X and Y). We would then have *n* −1 **=** 2 different decision functions **D**_*i*_ and the same **S**:

14 $$\begin{array}{cc}{\mathbf F}_i=\mathbf S+{\mathbf D}_i,&i=1,2.\end{array}$$

Because they share the same **S**, the shape differences among the {*F*_*i*_} (which can be described by their cumulants) depend only on the {**D**_*i*_}:

15 $$\begin{array}{cc}\kappa_m\left({\mathbf F}_i\right)-\kappa_m\left({\mathbf F}_j\right)=\kappa_m\left({\mathbf D}_i\right)-\kappa_m\left({\mathbf D}_j\right),&i=1,2;\;m=1,2,...\end{array}$$

Given a set of hypothesized decision processes, {**D**_*i*_}, we can therefore test for predicted differences between the cumulants of the decision processes by testing differences between the cumulants of the observed *PMF*s, {**F**_*i*_}. And, given the hypothesis of a set of decision processes, {**D**_*i*_}, estimates of cumulants of the distribution of sensory values can be obtained by subtraction:

16 $$\begin{array}{ccc}\kappa_m\left(\mathbf S\right)=\kappa_m\left({\mathbf F}_{\mathit i}\right)-\kappa_m\left({\boldsymbol D}_i\right),&i=1,2;&m=1,2,...\end{array}$$

*κ*_*m*_(**S**) should be the same across all of the {**F**_*i*_, **D**_*i*_} pairs, thus testing the hypothesis, and if the hypothesis is correct, we have estimates of the distribution of **S**, via its cumulants.^11^

## 5 A model with deterministic decisions

We consider first the generalization to a multiple-response procedure of the deterministic decision rule that was described by Eq. (4). Let *θ*_*i*_, *i* = 1, 2, ..., *n* − 1 be a set of ordered and fixed criteria on the continuum of the sensory variable **S** (which might be the arrival-time difference **A**_*y*_ − **A**_*x*_), with *θ*_*i*_* ≤ θ*_*i*+1_ for *i* **=** 1, 2, ..., *n* − 2. To simplify statements, define *θ*_0_ **=** −∞ and *θ*_*n*_ **=** ∞. Then the conventionally assumed decision rule for the rating procedure (e.g., Green & Swets, 1966, Section 2.4) can be stated as follows:

17 $$\begin{array}{ccc}\mathbf R=i&iff&\theta_{i-1}\leq\mathbf S<\theta_i,i=1,2,...,n,\end{array}$$

or

18 $$\begin{array}{ccc}\mathbf R>i&iff&S\geq\theta_i.\end{array}$$

Thus, if we define a set of *response-probability functions*, *h*_*i*_(*S*), each giving the probability of a particular response as a function of the sensory variable, **S** (possibly the arrival-time difference, **S = A**_*y*_ − **A**_*x*_),

19 $${h}_{i}\left(S\right)=Pr\left\{\mathbf{R}=i \;\right|\mathbf{S}=S\}, i=1, 2, . . . , n,$$

then the deterministic decision rule requires that

20 $$h_i\left(S\right)=\left\{\begin{array}{l}1,\theta_{i-1}\leq S<\theta_i\\0,\;elsewhere\end{array}\right.,\;i=1,2,\dots,n.$$

Now, by analogy with Eq. (3) we can define a set of decision functions, one associated with each response from **R** = 1 to **R** = n − 1:

21 $$D_i\left(S\right)=Pr\left\{\mathbf R>i\;\right|\mathbf S=S\},i=1,2,...,n-1.$$

Note that

22 $$D_i\left(S\right)=\sum_{j=i}^nh_j\left(S\right),-\infty<S<\infty,\;i=\text{1, 2},...,\;n-1.$$

As in the case of the *F*_*i*_, it follows from the definition of the *D*_*i*_ that they, also, are characterized by a dominance property:

23 $${D}_{i}\left(S\right)\ge {D}_{i+1}\left(S\right),-\infty <S<\infty , i=\text{1, 2},\dots,n-1.$$

By analogy with Eq. (4), it follows from Eqs. (20) and (22) that for the deterministic decisions model of Eq. (17) the *D*_*i*_ are all step functions,

24 $$D_i\left(S\right)=\left\{\begin{array}{c}0,S<\theta_i\\1,S\geq\theta_i\end{array}\right.,$$

so that the corresponding random variables are all constants. But from Eqs. (20) and (22),

25 $${\mathbf F}_i\left(d\right)=Pr\left\{\mathbf{S}\geq\theta_i\right\},$$

so that by analogy to Eq. (6), we can represent the **PMF**_*i*_ as follows:

26 $${\mathbf F}_i=\mathbf S+\theta_i,i=1,2,...,n-1.$$

Thus for the deterministic decisions model the **F**_*i*_ represent random variables that differ from each other only because they involve different additive constants θ_*i*_; in terms of the *F*_*i*_(*d*) we have

27 $${F}_{i}\left(d+{\theta }_{i}\right)={F}_{j}\left(d+{\theta }_{j}\right),$$

which shows that *for the deterministic decisions model the PMFs are parallel — i.e., differ only by translation on the d-axis.* That is, for *m* > 1, the *κ*_*m*_(*F*_*i*_) are the same for all *i*. Note that for temporal-order perception, this result does not depend on the distributions of the arrival latencies **L**_*x*_ and **L**_*y*_. Properties of the model are shown in Tables 5.1–5.4.

Perhaps our intuition that the PMFs in a family should be parallel is based on an implicit belief in the deterministic decisions model.

## 6 A general probabilistic decisions model

Generalizing further to nondeterministic decision rules (where the *D*_*i*_ defined in Eq. (21) are not all step functions) we have by analogy with Eq. (7),

28 $$\begin{array}{cc}{\mathbf F}_i=\mathbf S+\mathbf D_i,&i=1,...\;,n-1,\end{array}$$

with *D*_*i*_ defined^12^ as the distribution function of **D**_*i*_. And as it did earlier, cumulant additivity gives us *κ*_*m*_(**F**_*i*_) **=** *κ*_*m*_(**S**) + *κ*_*m*_(**D**_*i*_). Just as in the case of the general independent-channels model for the binary-choice experiment (Sternberg & Knoll, 1973, Section II.C), further specification of the *D*_*i*_ beyond the dominance property (Eq. 23) follows from particular models of the decision mechanism; three examples of such models are discussed in the sections below. But the formulation of the general model in Eq. (28) allows us to state the restriction on the decision functions *D*_*i*_ that is required if the PMFs, {*F*_*i*_}, are to be parallel: For the distributions of **F**_*i*_ and **F**_*j*_ to differ by translation only (so that **F**_*i*_ ≈ **F**_*j*_ + *K*), the distributions of **D**_*i*_ and **D**_*j*_ must differ by translation only.^13^ That is, *for the General Probabilistic Decisions Model, the F*_*i*_* are parallel on the d-axis if and only if the D*_*i*_* are parallel on the* **S** *axis.*^14^

## 7 A threshold model

In one of the simplest nondeterministic decision models, there is a threshold interval centered around **S =** 0, within which different **S** values cannot be discriminated from each other. (See Model 3 in Sternberg & Knoll, 1973, Section II.C.)^15^ Response probabilities are shown in Table 7.1.

**Table 7.1:** Threshold Model: Response Probabilities in five intervals along the **S** axis. In the threshold region [−*τ*, *τ*) the observer is assumed to guess, with *Pr*{**R =** 2} **=** 1 −* γ* and *Pr*{**R =** 3} **=*** γ*, where 0 ≤ *γ* ≤ 1.

**S** Interval	[−∞, *θ*_1_)	[*θ*_1_, − *τ*)	[−*τ*, *τ*)	[*τ*,*θ*_2_)	[*θ*_2_, +∞)
Response	**R** = 1	**R** = 2	**R** = 2 or 3	**R** = 3	**R** = 4
*h*_1_ **=** *Pr*{**R =** 1}	1	0	0	0	0
*h*_2_ **=** *Pr*{**R =** 2}	0	1	1 − *γ*	0	0
*h*_3_ **=** *Pr*{**R =** 3}	0	0	*γ*	1	0
*h*_4_ **=** *Pr*{**R =** 4}	0	0	0	0	1

In the model described in Table 7.1, there are four alternative responses, and the threshold lies within the interval on the **S** axis covered by the two middle responses, **R =** 2 and **R =** 3; within the threshold region [−*τ ≤* **S** < *τ*) these responses have probabilities1 – *γ* and *γ*, respectively, while outside the threshold region the deterministic decisions model applies. Consequences for the *D*_*i*_ distributions, probability functions, and cumulants are shown in Tables 7.2, 7.3, and 7.4:

**Table 7.2:** Threshold Model: Cumulative Distribution Functions of the {*D*_*i*_}

**S** Interval	[−∞, *θ*_1_)	[*θ*_1_, −* τ*)	[−*τ*, *τ*)	[*τ*,*θ*_2_)	[*θ*_2_, +∞)
*D*_1_ **=** *Pr*{**R** > 1}	0	1	1	1	1
*D*_2_ **=** *Pr*{**R** > 2}	0	0	*γ*	1	1
*D*_3_ **=** *Pr*{**R** > 3}	0	0	0	0	1

**Table 7.3:** Threshold Model: Probability Functions of the {*D*_*i*_}

Function	Value			
	*θ*_1_	*−τ*	*τ*	*θ*_2_
*g*_1_	1	0	0	0
*g*_2_	0	*γ*	1 − *γ*	0
*g*_3_	0	0	0	1

**Table 7.4:** Threshold Model: *D*_*i*_ Cumulants

Cumulant	Distribution		
	*D*_1_	*D*_2_	*D*_3_
*κ*_1_	*θ*_1_	(1 − 2* γ*)*τ*	*θ*_2_
*κ*_2_	0	4*γ* (1 −* γ*)*τ*^2^	0
	0	−8*γ* (1 −* γ*)(1 − 2*γ*)*τ*^3^	0
*κ*_3_			
	0	16(*γ* − 7*γ*^2^ + 12*γ*^3^ − 6*γ*^4^)*τ*^4^	0
*κ*_4_			

Note that the *D*_*i*_ distributions have all their probabilities at a small number of discrete values. For a discrete distribution, the “probability function”, or “probability mass function”, *g*_*i*_ (in Table 7.3) consists of the probability increments (or decrements) in the cumulative distribution function *D*_*i*_ associated with each of the values at which there is an increment (or decrement). The probability functions can be used for the calculation of cumulants.

As an example of the calculation of cumulants we show how *κ*_2_ (which is the same as the second moment) for the *D*_2_ distribution can be determined. For values −* τ* and *τ*, *g*_2_ gives us probabilities *γ* and 1 − *γ*, respectively. We first determine the mean, *m* of *g*_2_: *m* **=** *γ* (−*τ*) + (1 − *γ*)* τ* **=** (1 − 2* γ*)*τ*. For deviations from the mean, we then have −* τ* − *m* and *τ* − *m* with probabilities *γ* and 1 − *γ*, respectively. The second moment is then *γ* (−*τ* − *m*)^2^ + (1 − *γ*)(*τ* − *m*)^2^ **=** 4* γ* (1 − *γ*)*τ*^2^.

Examination of Tables 7.2 and 7.3 shows that whereas **D**_1_ and **D**_3_ are constants, with zero cumulants above the first, **D**_2_ has a two-point distribution with variance 4*τ* (1 − *γ*)*τ*^2^. *Hence, given Eq. *(28)*, F*_1_*, and F*_3_ *must be parallel, while the middle PMF, F*_2_*, must be flatter than the others,* with its variance larger by 4* γ* (1 − *γ*)*τ*^2^ than the variance of *F*_1_ and *F*_3_. Because *θ*_1_ < *θ*_2_, it follows that *κ*_1_(*F*_1_) < *κ*_1_(*F*_3_). Also, if guessing is unbiased (*γ*** =** 0.5), then *κ*_1_(*D*_2_) = *κ*_3_(*D*_2_) = 0, and if the **S** distribution is Gaussian as well, *κ*_3_(*F*_2_) = 0. If the guessing probabilities are biased, the signs of *κ*_1_(*D*_2_) and *κ*_3_(*D*_2_), which are opposite, depend on the direction of the bias. Because the decision process contributes nothing to them, the third cumulants of *F*_1_ and *F*_3_ (and *F*_2_ as well, if *γ* = 0.5) depend entirely on the contribution from **S**, and must therefore be equal; if it is plausible that the distribution of **S** is symmetric (as are the Gaussian and Laplace distributions; Kelber & Ulrich, 2024), the third cumulants should be zero.^16^*κ*_4_(*D*_2_) will be negative for 0.22 ≤ *γ* ≤ 0.78 (likely) and positive otherwise; if the distribution of **S** is Gaussian this will also be true of *κ*_4_(*F*_2_).

Appendix C provides examples of families of PMFs generated by the threshold model, and shows some of the effects of parameter changes.

## 8 A model where a confident and correct report of successiveness may be associated with an erroneous report of order

Here we consider implications of a “successiveness model”, in which the mechanisms subserving the perception of successiveness might be different and, to some extent, independent of those subserving the perception of order.^17^ Given that the perception of the order of two events requires discrimination and correct assignment of their identities (as pointed out by Hirsh & Sherrick, 1961), whereas the perception of successiveness probably does not, and given the findings of Hirsh (1959) of smaller frequency thresholds for successiveness than order perception in audition, the separation of these aspects of time perception seems reasonable to consider.^18^ Such a model is analogous to the one considered by Wickelgren (1969) for comparison of pitches, in which the degree of similarity between the two pitches is discriminated by a different mechanism from the one that discriminates which pitch is higher (see also Semal & Demany, 2006, who found that for some listeners, the frequency threshold for discriminating the direction of a pitch difference is much greater than the threshold for detecting that they differ.)

This model is suitable for the procedure used in the “joint sessions” by Allan (1975a), in which observers judged the relative offset times of a tone and a light and provided one of four pairs of judgments: “successive and tone first” (**R** = 1), “simultaneous and tone first” (**R** = 2), “simultaneous and light first” (**R** = 3), and “successive and light first” (**R** = 4).^19^ The body of Table 8.1 can be divided into four quadrants: In the upper left and lower right quadrants, the order response is correct; in the other quadrants, the order response is incorrect. Within the [−*τ*,* τ*) region, the observer is assumed to guess either **R** = 2 or **R** = 3, with probabilities that can (but need not) differ in the two halves of the region. As shown by the model specifications in the table, when −2*τ* ≤ **S** < −* τ* the observer can correctly and confidently judge *X* and *Y* to be successive (**R** = 1 or **R** = 4), while misperceiving their order (**R =** 4) on a fraction *γ* > 0 of trials, and similarly when *τ* ≤ **S** < 2*τ*. When −* τ* ≤ **S** <* τ*, the observer is sensitive to the sign of **S** (**R =** 3 more likely than **R** = 2, when **S** > 0), but the order is nonetheless misperceived (**R** = 2) on a fraction *λ* of trials. Because we shall be describing another “successiveness” model, based on a procedure with three response alternatives, we call this the “four-response successiveness model”.

**Table 8.1:** Four-Response Successiveness Model: Response Probabilities in six intervals along the **S = A**_*y*_ − **A**_*x*_ axis. (0 < *γ* < *λ* ≤ 0.5)

**S** Interval:	[−∞, −2*τ*)	[−2*τ*,−*τ*)	[−*τ*, 0)	[0, *τ*)	[*τ*, 2*τ*)	[2*τ*, +∞)
Response:	**R** = 1	**R** = 1 or 4	**R** = 2 or 3	**R** = 3 or 2	**R** = 4 or 1	**R** = 4
*h*_1_ = *Pr*{**R** = 1}	1	1 − γ	0	0	*γ*	0
*h*_2_ = *Pr*{**R** = 2}	0	0	1 − *λ*	*λ*	0	0
*h*_3_ = *Pr*{**R** = 3}	0	0	*λ*	1 − *λ*	0	0
*h*_4_ = *Pr*{**R** = 4}	0	*γ*	0	0	1 − *γ*	1

(We have used response-probability functions *h*_*i*_ that are constant within intervals on the **S** axis as well as intervals that are of equal width (*τ*) for simplicity; in a more general model both these restrictions might be relaxed.)

**Table 8.2:** Four-response Successiveness Model: Cumulative Distribution Functions of the {*D*_*i*_}

**S** Interval	[−∞, − 2*τ*)	[−2*τ*, −*τ*)	[−*τ*, 0)	[0,*τ*)	[*τ*, 2*τ*)	[2*τ*, +∞)
*D*_1_ **=** *Pr*{**R** > 1}	0	*γ*	1	1	1 − *γ*	1
*D*_2_ **=** *Pr*{**R** > 2}	0	*γ*	*λ*	1 − *λ*	1 − *γ*	1
*D*_3_ **=** *Pr*{**R** > 3}	0	*γ*	0	0	1 − *γ*	1

Unlike the *D*_*i*_ described in Tables 5.2 and 7.2, respectively, not all the *D*_*i*_ generated by the present model and shown in Table 8.2 are nondecreasing functions; instead, *D*_1_ and *D*_3_ are nonmonotonic and as a result cannot correspond to actual random variables.^20^ The distribution to which *D*_1_ corresponds, for example, included in Table 8.2, would have negative probability (−*γ*) at **S** = *τ*. Nonetheless for present purposes we can apply the usual operations to derive the cumulants of the {*D*_*i*_}, listed in Table 8.4, that combine additively with those of **S** to produce the corresponding cumulants of the PMFs {*F*_*i*_}, as implied by Eq. (28).

**Table 8.3:** Four-Response Successiveness Model: Probability Functions of the {*D*_*i*_}

	Value				
Function	−2*τ*	−*τ*	0	*τ*	2*τ*
*g*_1_	*γ*	1 − *γ*	0	−*γ*	*γ*
*g*_*2*_	*γ*	*λ* − *γ*	1 − 2*λ*	*λ* − *γ*	*γ*
*g*_*3*_	*γ*	− *γ*	0	1 – *γ*	*γ*

**Table 8.4:** Four-Response Successiveness Model: *D*_*i*_ Cumulants

Cumulant		Distribution	
	*D*_1_	*D*_2_	*D*_3_
*κ*_1_	*−τ*	0	*τ*
	6*γτ*^2^	(6*γ +* 2*λ*)*τ*^2^	6*γτ*^2^
*κ*_2_			
	18*γτ*^3^	0	−18*γτ*^3^
*κ*_3_			
	(66*γ −* 108*γ*^2^)*τ*^4^	(30*γ +* 2*λ* − 108*γ*^2^ − 12*λ*^2^ − 72*γλ*)*τ*^4^	(66*γ* − 108*γ*^2^)*τ*^4^
*κ*_4_

**Table 8.5:** Four-Response Successiveness Model: Cumulant Relations

	Relation	Difference
(1)	*κ*_1_(*F*_1_) < *κ*_1_(*F*_2_)	*τ*
(2)	*κ*_1_(*F*_2_) < *κ*_1_(*F*_3_)	*τ*
(3)	*κ*_2_(*F*_1_) < *κ*_2_(*F*_2_)	2*λτ*^2^
(4)	*κ*_2_(*F*_2_) > *κ*_2_(*F*_3_)	2*λτ*^2^
(5)	*κ*_3_(*F*_1_) > *κ*_3_(*F*_2_)	18*γτ*^3^
(6)	*κ*_3_(*F*_2_) > *κ*_3_(*F*_3_)	18*γτ*^3^
(7)	2*κ*_1_(*F*_2_) **=** *κ*_1_(*F*_1_) + *κ*_1_(*F*_3_)	0
(8)	*κ*_2_(*F*_1_) **=** *κ*_2_(*F*_3_)	0
(9)	2*κ*_3_(*F*_2_) **=** *κ*_3_(*F*_1_) + *κ*_3_(*F*_3_)	0
(10)	*κ*_4_(*F*_1_) **=** *κ*_4_(*F*_3_)	0

The results in Table 8.4 show that *D*_2_ has a greater variance (by 2*λτ*^2^) than *D*_1_ or *D*_3_, whose variances are equal, which means that *F*_2_ has a greater variance (by 2*λτ*^2^) than *F*_1_ or *F*_3_, whose variances are equal. They also show that whereas *D*_2_ is symmetric, *D*_1_ is positively skewed (third cumulant = 18*λτ*^3^) and *D*_3_ is negatively skewed by the same amount. If we assume that the **S** = **A**_*y*_ − **A**_*x*_ distribution is symmetric, which is often plausible and for which there is good evidence from temporal-order judgments (Kelber & Ulrich, 2024), this statement also applies to the PMFs, *F*_1_, *F*_2_, and *F*_3_.^21^ Without this assumption, the model implies that *κ*_3_(*F*_1_) > *κ*_3_(*F*_2_) > *κ*_3_(*F*_3_), and that the magnitude of each of the two differences is 18*γτ*^3^. The relations among the cumulants of *F*_1_, *F*_2_, and *F*_3_ are summarized by the six inequalities and four equalities in Table 8.5. In that table, the equality of the differences of (1) and (2) is captured by (7), the equality of the differences of (3) and (4) is captured by (8), and the equality of the differences of (5) and (6) is captured by (9).

Appendix C provides examples of PMFs generated by the Four-Response Successiveness Model, and shows some of the effects of parameter changes. Note that for several sets of parameter values, *F*_1_ and *F*_3_, and, less frequently, *F*_2_, are nonmonotonic.

## 9 A Three-Response Successiveness Model

A model that was recently proposed and tested successfully by Kelber and Ulrich (2024) is a variant of the Four-Response Successiveness Model that accommodates experiments with three alternative responses: “*Y first*” (**R** = 1), “*X*, *Y simultaneous*” (**R** = 2), and “*X first*” (**R** = 3). They called it the “two-threshold model”, and found that it was the best of the three models they tested in accounting for the PMFs for eight substantial data sets from temporal-order experiments. It generates two PMFs, similar to Allan’s *F*_1_ and *F*_3_. In this model, the **S** axis is divided into five intervals, shown in Tables 9.1 and 9.2.

**Table 9.1:** Three-Response Successiveness Model. Response Probabilities in five intervals along the **S = A**_*y*_ −** A**_*x*_ axis. (0 ≤ *α* ≤ *β*; 0 ≤ *γ* **≤** 0.5)

**S** Interval	[−∞, −*β*)	[−*β*, −*α*)	[−*α*, *α*)	[*α*, *β*)	[*β*, +∞)
Response	**R** = 1	**R** = 1 or 3	**R** = 2	**R** = 1 or 3	**R** = 3
*h*_1_ **=** *Pr*{**R =** 1}	1	1 – *γ*	0	1 – *γ*	0
*h*_2_ **=** *Pr*{**R =** 2}	0	0	1	0	0
*h*_3_ **=** *Pr*{**R =** 3}	0	*γ*	0	*γ*	1

**Table 9.2:** Three-Response Successiveness Model. Cumulative Distribution Functions of the {*D*_*i*_}

**S** Interval	[−∞,−*β*)	[−*β*, −* α*)	[−*α*, *α*)	[*α*, *β*)	[*β*, +∞)
*D*_1_ **=** *Pr*{**R** > 1}	0	*γ*	1	*γ*	1
*D*_2_ **=** *Pr*{**R** > 2}	0	*γ*	0	*γ*	1

As in the four-response successiveness model, the probability functions *g*_*i*_ (shown in Table 9.3) contain some values with negative probabilities.

**Table 9.3:** Three-Response Successiveness Model. Probability Functions of the {*D*_*i*_}

	Value			
Function	*− β*	*− α*	*α*	*β*
*g*_1_	*γ*	1 *− γ*	*−* (1 *− γ*)	1 *− γ*
*g*_*2*_	*γ*	*−γ*	*γ*	1 *− γ*

Because cumulant calculations for the general model lead to complex expressions, we decided to assume unbiased guessing (*γ* = 0.5), with the simpler results shown in Table 9.3. For temporal-order judgments, when Kelber & Ulrich (2024) fitted this model to the eight data sets they considered, they found that, weighted by the number of observers, the mean of the values they obtained for $\widehat{\gamma }$ is 0.506.

The cumulants of *D*_1_ and *D*_2_ for the Three-Response Successiveness Model have properties that are similar to those of the cumulants of *D*_1_ and *D*_3_, respectively, for the four- response model shown in Table 8.4: The values of *κ*_1_ are equal in magnitude, but opposite in sign, with the value for *D*_1_ negative; the values of *κ*_2_ are equal; the values of *κ*_3_ are equal in magnitude, but opposite in sign, with the value for *D*_1_ positive; and the values of *κ*_4_ are equal.

Not surprisingly, the Three-Response Successiveness Model implies fewer cumulant equalities and inequalities (Table 9.5) than the Four-Response Successiveness Model (Table 8.5).

Appendix C provides examples of PMFs generated by the Three-Response Successiveness Model, and shows some of the effects of parameter changes. Note that as for the four-response model, a number of the PMFs are not monotonic.

## 10 A model with fluctuating criteria

Consider a decision model involving criteria *θ*_*i*_ on the **S** = **A**_*y*_ − **A**_*x*_ axis, like the Deterministic Decisions Model discussed in Section 5, but which permits the criteria to fluctuate from trial to trial so that they become random variables **H**_*i*_. (For binary-choice data, such a model with a single criterion was considered in Section 2). We assume that on each trial the ordering of criteria, *θ*_*i*_ ≤ *θ*_*i*+1_, assumed in Section 5, is preserved. This implies that for any **S** = **A**_*y*_ − **A**_*x*_, *Pr*{**H**_*i*+1_ ≤ *S*} ≤ *Pr*{**H**_*i*_ ≤ *S*}. Furthermore, since *Pr*{**H**_*i*_ ≤ *S*} **=** *Pr*{**R** > *i* | **A**_*y*_ − **A**_*x*_ **=** *S*} **=** *D*_*i*_(*S*), we see that not only can the decision function *D*_*i*_ be identified as the distribution function of the criterion **H**_*i*_ (which, incidentally, requires it to be a nondecreasing function), so that **D**_*i*_ and **H**_*i*_ are the same, but also that the dominance property (Eq. 23) required of the decision functions is guaranteed. The criterion distributions may overlap so long as the “amount” of overlap is not so great as to violate the dominance property. (If the distributions do overlap, however, the requirement of criterion ordering on every trial implies that the **H**_*i*_ cannot fluctuate independently.)

It should be noted that any model with nondecreasing *D*_*i*_, can be regarded as equivalent to a model with multiple fluctuating criteria, if it is reasonable to identify the *D*_*i*_ as criterion distributions. (The models described in Sections 8 and 9 cannot be equated in this way, because their *D*_*i*_ are not monotonic functions).

Having identified the *D*_*i*_ with the distributions of fluctuating criteria, we can immediately state the conditions for parallel PMFs: *In a model with two or more fluctuating criteria the F*_*i*_* are parallel if and only if the criterion distributions are identical except for location.* That is, if we define ${\mathbf{H}}_{i}^{*}=\mathbf{H}_{i}-E\left({\mathbf{H}}_{i}\right)$ to be the distribution of the *i*^th^ criterion adjusted for zero mean, parallel *F*_*i*_ requires that

29 $${\mathbf{H}}_{i}^{*}\approx {\mathbf{H}}_{j}^{*},1\le i,j\le n-1.$$

How likely are the distributions of multiple criteria to differ only in mean? We are not aware of any discussion of this question, and here we mention only two of the considerations that might bear on it. The distributional identity above requires, for example, that the criterion variance be the same for extreme criteria as for middle-range criteria. From a Weber-law viewpoint, on the other hand, one might expect the standard deviation of the criterion distribution to increase linearly with |*d*|, or with |*d* − *PSS*| (where PSS is the point of subjective equality or simultaneity). The result would be a generalized bidirectional Weber law: *the PMFs associated with more extreme responses would be flatter.*

Perhaps a more compelling argument for constraining the criterion distributions arises from the inherent symmetry of the decision aspects of the experimental paradigm: Given a pair of stimuli, their assignment to *X* and *Y* is arbitrary. A simple way of describing the consequence is that the series of density functions of the criteria **H**_1_, **H**_2_, ... on the **S = A**_*y*_ − **A**_*x*_ axis (i.e., viewed from the low *A*_*y*_ − *A*_*x*_ end) should have the same sequence of shapes as the corresponding series on the **A**_*x*_ − **A**_*y*_ axis (i.e., viewed from the opposite end). This implies that the density function of ${\mathbf{H}}_{i}^{*}$ should be the reflection of the density function of ${\mathbf{H}}_{n-i}^{*}$, or

30 $$\begin{array}{cc}\mathbf H_i^\ast\approx-\mathbf H_{n-i}^\ast,&i=1,2,...\;,n-1.\end{array}$$

But in order that the *F*_*i*_ differ by translation only, Eq. (29) must also be satisfied. Eqs. (29) and (30) together imply that the criterion distributions are symmetric about their means: ${\mathbf{H}}_{i}^{*}$ ≈ −${\mathbf{H}}_{i}^{*}$, *i* = 1, 2, ... , *n −* 1. Conversely, given Eq. (30), *any asymmetry in the criterion distributions will produce shape differences among the F*_*i*_.

**Fig. 1:**
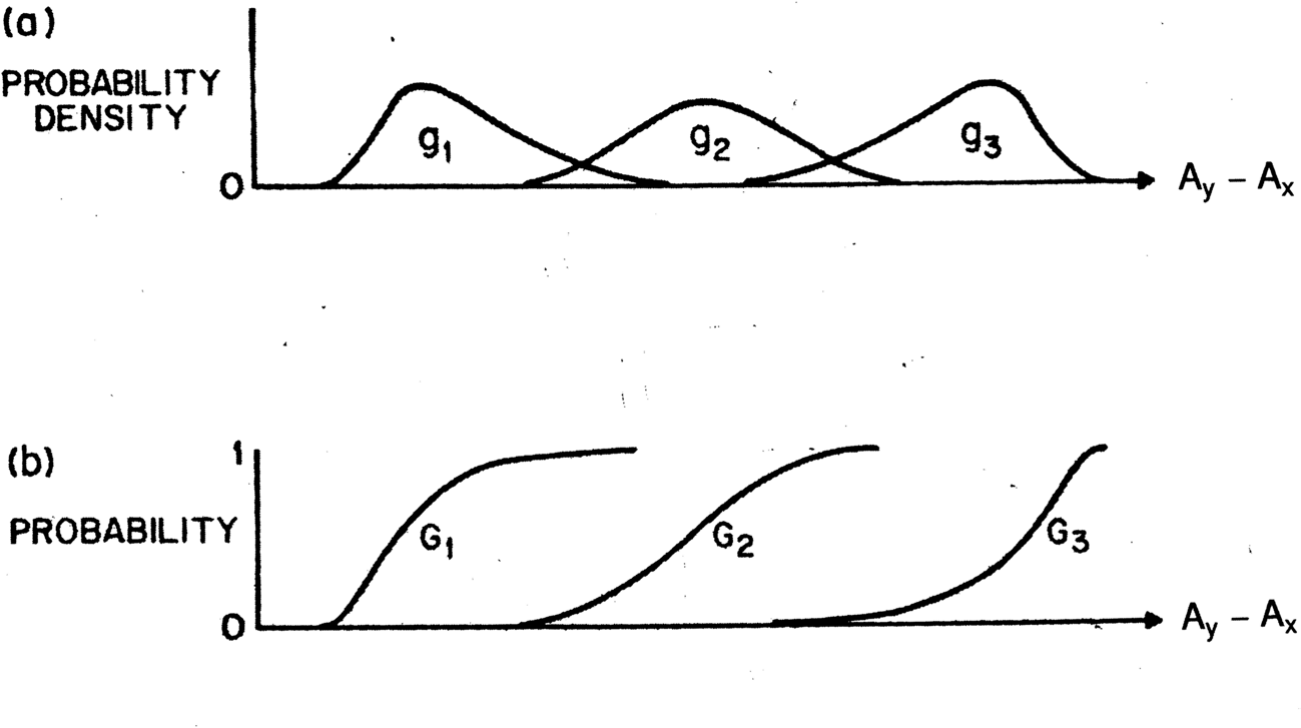
Hypothetical criterion distributions with (**a**) density functions that may be asymmetric in shape, but satisfy the requirement (imposed by experimental symmetry) that the density g_1_ of B_1_^*^ is the reflection of the density g_3_ of B_3_^*^ and (**b**) the nonparallel distribution functions to which they correspond

Suppose, for example, that the distribution of the lowest criterion is skewed toward high **A**_*y*_ − **A**_*x*_ values, as illustrated in Fig. 1. Then the argument from symmetry of the experiment implies that the distribution of the highest criterion should be skewed toward low **A**_*y*_ − **A**_*x*_ values (i.e., high **A**_*x*_ − **A**_*y*_ values). The consequence of the arrangement in the figure is that the PMFs {*F*_*i*_} would be more negatively skewed with larger *i*. In Section 5 we showed that observation of such shape differences among the *F*_*i*_ would require rejection of a model with fixed criteria; here we have shown that they are consistent with a model in which the criteria are permitted to fluctuate from trial to trial.

## 11 Families of PMFs from three experiments

We have described implications of the models for cumulants of the PMFs in Table 7.4 for the Threshold Model, in Tables 8.4 and 8.5 for the Four-Response Successiveness Model, and in Tables 9.4 and 9.5 for the Three-Response Successiveness Model. The predictions that specify equalities or directions of inequalities of PMF cumulants are independent of parameter values, but nonetheless permit models to be falsified. However, parameter values would enable better model tests, including the magnitudes of cumulant inequalities and the test specified by Eq. (11) that could be applied to all of the multiple-response models.

**Table 9.4:** Three-Response Successiveness Model with Unbiased Guessing (*γ* **=** 0.5): *D*_*i*_ Cumulants

Cumulant	Distribution	
	*D*_1_	*D*_2_
*κ*_1_	−*α*	*α*
*κ*_2_	*β*^2^ *− α*^2^	*β*^2^ *− α*^2^
*κ*_3_	3*α* (*β*^2^ *− α*^2^)	−3*α*(*β*^2^ *− α*^2^)
*κ*_4_	2(*β*^2^ *− α*^2^)(5*α*^2^ *− β*^2^)	2(*β*^2^ *− α*^2^)(5*α*^2^ *− β*^2^)

**Table 9.5:** Three-Response Successiveness Model with Unbiased Guessing (*γ* = 0.5): Cumulant Relations

	Relation	Difference
(1)	*κ*_1_(*F*_1_) < *κ*_1_(*F*_2_)	2*α*
(2)	*κ*_3_(*F*_1_) > *κ*_3_(*F*_2_)	6*α*(*β*^2^ – *α*^2^)
(3)	*κ*_2_(*F*_1_) **=** *κ*_2_(*F*_2_)	0
(4)	*κ*_4_(*F*_1_) **=** *κ*_4_(*F*_2_)	0

Further development of the ideas in this paper should therefore include development and testing of methods of parameter estimation. This could be done by using the model simulation programs (Appendix B), and fitting either the PMFs or their cumulants to the observations.^22^ To exemplify the inferential possibilities of the multiple-PMF method using only parameter-independent tests, we provide, as examples, families of PMFs from three experimental procedures that have been used in the study of temporal-order perception, procedures that used three or four ordered response alternatives. Evaluation of models using these families of PMFs involves comparison of the PMF shapes, as described by their cumulants, to the shapes and relations among shapes required by the models.

There are many studies that have used three ordered responses, yielding two PMFs, including five of the substantial data sets considered by Kelber & Ulrich (2024). In what follows we consider data from the study by van Eijk, et al. (2008). However, despite their possibly greater utility, we know of remarkably few psychophysical experiments of any kind that use four ordered responses and yield three PMFs; the only ones we know of are one described by Eijkman et al. (1966), Experiment 1 in Keane, et al. (2015), and those described in the two reports by Allan (1975a, 1975b), using the procedures described in Section 1. In each of Allan’s experiments we selected data from the observer whose PMFs come closest to spanning the full range of proportions from zero to one. Even these PMFs are not ideal for our purposes, however, as several fail to span the full range, perhaps caused by lapses of attention (and associated guessing) for which we have not corrected.

**Fig. 2:**
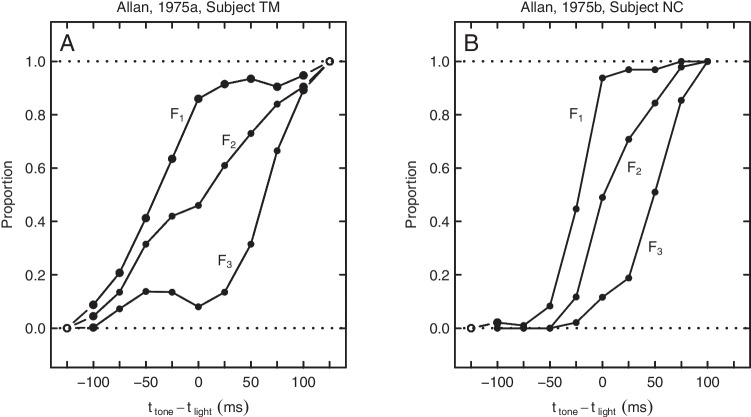
Two families of psychometric functions. Open circles mark extended values

In one of the procedures used by Allan (1975a), described in Section 1, observers judged the offset times of a tone and a light, making a successiveness judgment (“simultaneous” or “successive”) as well as an order judgment. The four combined judgments were, then, “successive and tone first” (**R =** 1), “simultaneous and tone first” (**R =** 2), “simultaneous and light first” (**R =** 3), and “successive and light first” (**R =** 4). **R =** 1 and **R =** 4 can be thought of as high confidence order judgments, and **R =** 2 and **R =** 3 as low confidence order judgments. These four responses permit defining three PMFs, *F*_1_ **=** *Pr*{**R** > 1}, *F*_2_ **=** *Pr*{**R** > 2}, and *F*_3_ **=** *Pr*{**R** > 3}, each giving a response proportion as a function of the stimulus, *d*_*i*_ (tone offset time − light offset time). The values of *F*_1_, *F*_2_, and *F*_3_ for Observer TM are shown in Table 11.1, for each offset-time difference, *d*_*i*_. Note that neither *F*_1_ nor *F*_2_ is monotonic. To arrange that the proportions { *p*_*i*_} span the full range from zero to one, they have to be extended; the extended values are shown in boldface in Tables 11.1 and 11.2, and by open circles in Fig. 2.^23^

**Table 11.1:** Family 1. Allan (1975a), Observer TM (Panel A of Figure 1)

d (ms)	(−125)	−100	−75	−50	−25	0	+25	+50	+75	+100	(+125)
Trials		96	96	96	96	384	96	96	96	96	
*F*_1_	**0.000**	0.087	0.208	0.413	0.635	0.860	0.915	0.935	0.905	0.948	**1.000**
*F*_2_	**0.000**	0.045	0.135	0.315	0.420	0.460	0.610	0.730	0.840	0.905	**1.000**
*F*_3_	**0.000**	0.002	0.073	0.138	0.135	0.080	0.135	0.315	0.665	0.893	**1.000**

**Table 11.2:** Family 2. Allan (1975b), Observer NC (Panel B of Figure 2)

d (ms)	(−125)	−100	−75	−50	−25	0	+25	+50	+75	+100
Trials		32	32	32	32	128	32	32	32	32
*F*_1_	**0.000**	0.021	0.010	0.083	0.447	0.938	0.969	0.969	1.000	1.000
*F*_2_		0.000	0.000	0.000	0.117	0.490	0.708	0.844	0.979	1.000
*F*_3_		0.000	0.000	0.000	0.021	0.116	0.188	0.510	0.854	1.000

In the procedure used in a second study by Allan (1975b), also mentioned in Section 1, observers judged the order of the offset times of a light and a tone, and also made a two-level confidence judgment (“certain” or “uncertain”). The four responses were therefore “tone first and certain” (**R =** 1), “tone first and uncertain” (**R =** 2), “light first and uncertain” (**R =** 3), and “light first and certain” (**R =** 4). Again, these permit defining three PMFs, *F*_1_ **=** *Pr*{**R** > 1}, *F*_2_ **=** *Pr*{**R** > 2}, and *F*_3_ **=** *Pr*{**R** > 3}, each as a function of the time difference, *d*_*i*_. The values of *F*_1_, *F*_2_, and *F*_3_ for Observer NC are shown in Table 11.2, and plotted in Panel B of Fig. 2. In this case extension was needed only for *F*_1_.

Estimated cumulants of these two families of PMFs are provided in Tables 11.3 and 11.4, respectively, determined by the method described in Appendix A.^24^ Where a PMF has been extended, Tables 11.3 and 11.4 provide cumulant estimates for PMFs with (${F}_{i}^{*}$) and without (*F*_*i*_) the extension.

**Table 11.3:** Family 1. Allan 1975a Observer TM: Estimated Cumulants (with bootstrap standard deviation estimates)

	*F*_1_	${F}_{1}^{*}$	*F*_2_	${F}_{2}^{*}$	*F*_3_	${F}_{3}^{*}$
*κ*_1_	−31 (4)	−35 (3)	−5 (4)	1 (3)	40 (4)	52 (2)
*κ*_2_	1385 (383)	2903 (303)	2689 (409)	4488 (323)	2610 (277)	2927 (295)
*κ*_3_/10^2^	347 (409)	1736 (378)	39 (181)	250 (276)	−1837 (334)	−2485 (378)
*κ*_4_/10^4^	296 (341)	1249 (351)	−717 (336)	−2279 (515)	690 (292)	1516 (348)

**Table 11.4:** Family 2. Allan 1975b Observer NC: Estimated Cumulants (with bootstrap standard deviation estimates)

	*F*_1_	${F}_{1}^{*}$	*F*_2_	*F*_3_
*κ*_1_	−21 (3)	−23 (3)	9 (3)	45 (3)
*κ*_2_	523 (195)	685 (184)	1106 (195)	972 (162)
*κ*_3_/10^2^	161 (88)	44 (156)	184 (88)	−202 (102)
*κ*_4_/10^4^	91 (74)	187 (110)	−72 (74)	8 (62)

As an example of three-response data we chose results of the experiment by van Eijk, et al. (2008). Each observer in this experiment was run in two conditions: In one, with eleven observers, the two stimuli were a flash and a click; In the other (with the same 11 observers together with a twelfth) the stimuli were the image of a ball bouncing and a corresponding impact sound. In both of these conditions, the time difference ranged from − 350 to 350 ms with intervals of 50 ms. We found that for eight of the eleven pairs of PMFs in the flash-click condition, the proportions associated with − 350 and 350 ms were *P*(−350) ≤ 0.02 and *P*(350) ≥ 0.98, respectively, whereas this was true for only six of the 12 pairs of PMFs in the bouncing-ball condition; we thus decided to consider only the cumulants of the eight pairs of PMFs from the flash-click condition, after extending them by setting proportions of zero and one at − 400 and 400 ms, respectively. Mean estimated cumulants for these PMFs are provided in Table 11.5.

## 12 Some tests of the four-response models

Here we consider the models described in Sections 5, 7, and 8 in relation to the first two families of PMFs described in Section 11.

*According to the Deterministic Decisions Model* (Section 5):

- The PMFs in a family must be parallel. As shown, for example, by differences among the values of *κ*_3_ in Tables 11.3 and 11.4 for *F*_1_ and *F*_3_, this is clearly false for both families.

*According to the Threshold Model* (Section 5):

- *κ*_2_(*F*_2_) must be larger than both *κ*_2_(*F*_1_) and *κ*_2_(*F*_3_). This is true for both Families 1 and 2 (Tables 11.3 and 11.4), whether we consider *F*_1_, *F*_2_, and *F*_3_ or $F_1^\ast$, $F_2^\ast$, $F_3^\ast$ in Table 11.3 and *F*_1_ or $F_1^\ast$ in Table 11.4.
- *F*_1_ and *F*_3_ must be parallel. This is falsified by Families 1 and 2. For example, *F*_1_ and *F*_3_ are skewed in opposite directions (Tables 11.3 and 11.4).
- If the **S** distribution is symmetric, which is plausible (Kelber & Ulrich, 2024), then we must have *κ*_3_(*F*_1_) **=** *κ*_3_(*F*_3_) **=** 0, which is falsified by Families 1 and 2 (Tables 11.3 and 11.4).

**Table 11.5:** Mean Estimated Cumulants (± *se*. *m*) of *F*_1_ and *F*_2_ for Eight Observers in the Flash-Click Condition of van Eijk et al. (2008)

Measure	*F*_1_	*F*_2_
$\overline{{\kappa }_{1}}/{10}^{2}$	−0.70 ± 0.11	1.33 ± 0.19
$\overline{{\kappa }_{2}}/{10}^{3}$	9.65 ± 1.46	9.90 ± 1.42
$\overline{{\kappa }_{3}}/{10}^{6}$	1.76 ± 0.55	−1.73 ± 0.64
$\overline{{\kappa }_{4}}/{10}^{8}$	7.95 ± 2.30	7.54 ± 2.30

The values in Table 12.1 are the differences associated with the inequalities and equalities of Table 8.5. For example, for each subject, the value associated with the first inequality, *κ*_1_(*F*_1_) < *κ*_1_(*F*_2_), is the difference *κ*_1_(*F*_2_) − *κ*_1_(*F*_1_); the standard deviation (SD) of this difference is based on the bootstrap SD estimates of its components in Table 11.3 or 11.4

**Table 12.1:** Four-Response Successiveness Model: Evaluation of the Cumulant Relations of Table 8.5. Quantities in the Value (SD) columns have multipliers 10^2^ (rows 5, 6, and 9) and 10^4^ (row 10). Where a PMF was extended, the cumulants used for this analysis are based on the extended PMF, but the conclusions do not depend on this choice

	Observer TM		Observer NC	
	Value (SD)	Ratio	Value (SD)	Ratio
(1)	36 (4.2)	8.6	32 (4.2)	7.6
(2)	51 (3.6)	14.2	36 (4.2)	7.6
(3)	1585 (443)	3.6	421 (268)	1.6
(4)	1561 (437)	3.6	134 (254)	0.5
(5)	1486 (468)	3.2	−140 (179)	−0.8
(6)	2735 (468)	5.8	386 (135)	2.9
(7)	−15 (7)	2.1	−4 (5)	−0.8
(8)	25 (423)	0.1	287 (245)	1.2
(9)	1249 (768)	1.6	526 (206)	2.6
(10)	267 (494)	0.5	−179 (126)	−1.4

*According to the Four-Response Successiveness Model *(Section 8):

- Table 12.1 shows that the six inequalities in Table 8.3 are all satisfied by the cumulants for Observer TM. And all the inequalities except (5) are satisfied by the cumulants for Observer NC.
- The variance of *F*_2_ is greater than that of *F*_1_ or *F*_3_. This is true for both Families 1 and 2. (Tables 11.3 and 11.4.)
- if we assume that the distribution of **S** is symmetric (which is plausible; Kelber & Ulrich, 2024): *F*_1_ should be positively skewed (*κ*_3_ > 0) and *F*_3_ should be negatively skewed (*κ*_3_ < 0). This is true for both Families 1 and 2. (Tables 11.3 and 11.4).
- Again, assuming the symmetry of the **S** distribution, *F*_2_ should be symmetric (i.e., *κ*_3_(*F*_2_) = 0). This is better supported by the data for Observer TM [*κ*_3_(*F*_2_) = 250; SD = 276] than by the data for Observer NC [*κ*_3_(*F*_2_) = 184; SD = 88].

Based on these limited and imperfect data, the Four-Response Successiveness Model is promising

## 13 Some tests of the three-response model

The results in Table 13.1 show that the data convincingly satisfy the two inequalities and the two equalities in Table 9.5, consistent with the Three-Response Successiveness Model. This should not be surprising, as these data were shown by Kelber and Ulrich (2024), using a different approach, to be consistent with that model. The left side of Table 13.2 provides information about the distribution of sensory values in the Flash-Click condition of van Eijk et al. (2008). The absence of significant differences between cumulants of the two estimates of that distribution, shown on the right side of Table 13.2, confirms the expectation from Eq. (16), and thus supports the hypothesized decision process embodied in **D**_1_ and **D**_2_.

**Table 13.1:** Mean Differences between Cumulants of *F*_1_ and *F*_2._ for Eight Observers in the Flash-Click Condition of Van Eijk et al.(2008)

	Measure	*κ*_*r*_(*F*_2_) − *κ*_*r*_(*F*_1_)	(SE)	*p*-value
(1)	*κ*_1_/10^2^	2.04	(0.26)	< 0.0001
(2)	$\overline{{\kappa }_{3}}/{10}^{6}$	−3.49	(1.09)	0.015
(3)	$\overline{{\kappa }_{2}}/{10}^{3}$	0.26	(1.53)	0.87
(4)	$\overline{{\kappa }_{4}}/{10}^{8}$	−0.41	(1.46)	0.79

**Table 13.2:** Mean cumulants of Estimated Sensory-Value Distributions for Eight Observers in the Flash-Click Condition of Van Eijk et al.(2008). The left side of the table shows the means $\left[\kappa_r\left({\widehat{\mathbf S}}_1\right)+\kappa_r\left({\widehat{\mathbf S}}_2\right)\right]/2$; the right side shows the differences, *κ*_*r*_ $\left({\widehat{\mathbf{S}}}_{2}\right)$ − *κ*_*r*_ $\left({\widehat{\mathbf{S}}}_{1}\right)$

Measure	Mean	(SE)	*p*-value	Difference	(SE)	*p*-value
*κ*_1_	32.11	(6.54)	0.002	−1.51	(1.09)	0.21
*κ*_2_/10^3^	5.74	(0.58)	<0.0001	0.40	(0.78)	0.62
*κ*_3_/10^5^	0.17	(4.89)	0.97	−8.74	(10.95)	0.45
*κ*_4_/10^8^	4.24	(1.51)	0.026	0.49	(1.17)	0.69

## 14 Conclusions

Much can be gained at small cost by enriching the response alternatives in a psychophysical experiment: Relations among the cumulants of the resulting family of PMFs can be highly informative. To exemplify the inferential possibilities, we described a set of models for judgments of stimulus differences, inspired by judgments of stimulus time differences (temporal order), and determined their implications. We then used estimates of the cumulants of the members of three families of observed PMFs to test the models.

Given their effectiveness it is perhaps surprising that these tests were nonparametric: For the tests of each model, the cumulant properties that we used are independent of the model’s parameter values. Additional tests would be possible with parameter estimates.

In answer to the questions with which we began this paper, our intuition that the members of a PMF family should be parallel would be explained if, for example, we believed that the deterministic decisions model (Section 5) is valid. And if the PMFs are not parallel (as is the case), we have seen that much can be learned from differences among the spreads and shapes of family members, which enabled us to evaluate the other models we have described.

Some of our findings about the models include the following:

1. For the Deterministic Decisions Model, the PMFs {*F*_*i*_} are parallel — i.e., they differ only by translation on the stimulus axis.
2. For the General Probabilistic Decisions Model, the {*F*_*i*_} are parallel on the stimulus axis if and only if the decision functions *D*_*i*_ are parallel on the **S** axis.
3. For the Threshold Model with four ordered response categories, the middle PMF is flatter than the others, which are parallel.
4. For a Model with Fluctuating Criteria, the {*F*_*i*_} are parallel if and only if the criterion distributions are identical except for location.
5. For the Four-Response Successiveness Model, where successiveness can be accurately discriminated while order may not be and there are four ordered response categories, *F*_1_ and *F*_3_ have equal variances and, if we assume that the **S** distribution is symmetric, they are skewed (positively and negatively, respectively) by the same amount, while *F*_2_ has greater variance, but is symmetric.
6. For the Three-Response Successiveness Model (the “two-threshold model”; Kelber & Ulrich, 2024), the properties of *F*_1_ and *F*_2_ are similar to those of *F*_1_ and *F*_3_ of the four- response model: They have equal variances, and, if we assume that the **S** distribution is symmetric, they are skewed (positively and negatively, respectively) by the same amount.

Based on the observed PMF families we considered, and among the models we tested quantitatively, and especially given the findings by Kelber and Ulrich (2024), it is the Four-Response Successiveness Model and its three-response variant, similar to a model proposed for pitch perception by Wickelgren (1969), that seems the most promising.

These findings depend on treating each PMF as the distribution function of the sum of two stochastically independent random variables: one that represents the sensory difference (the arrival-time difference, for example), and another that represents the decision process. Model evaluations made use of the relations among the first four cumulants of the PMFs within a family, together with the cumulant-additivity property for sums of stochastically independent random variables.

## Figures and Tables

**Fig. C1:**
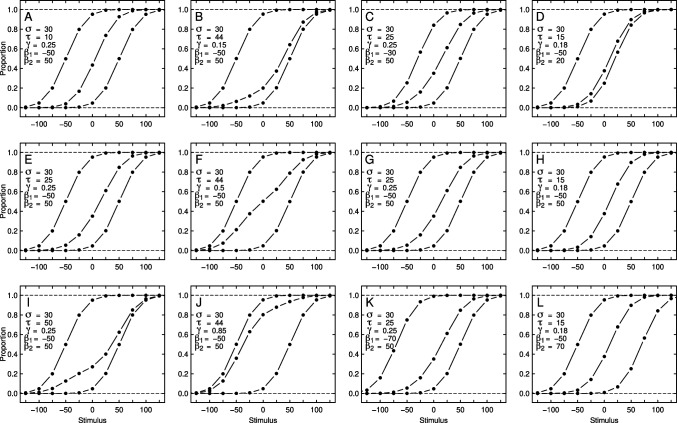
Threshold Model: Simulated Psychometric Functions

**Fig. C2:**
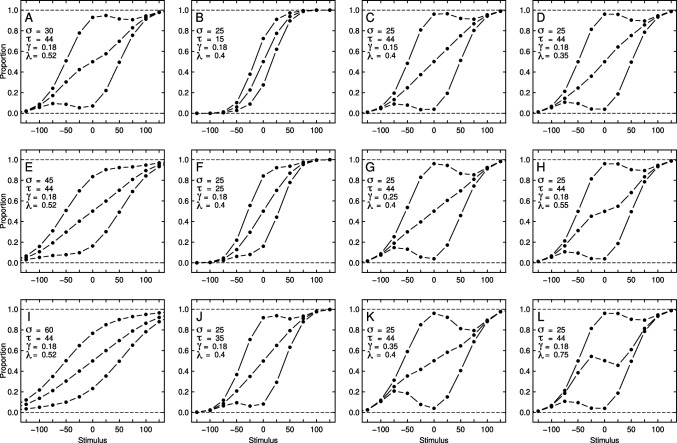
Four−Response Successiveness Model: Simulated Psychometric Functions

**Fig. C3:**
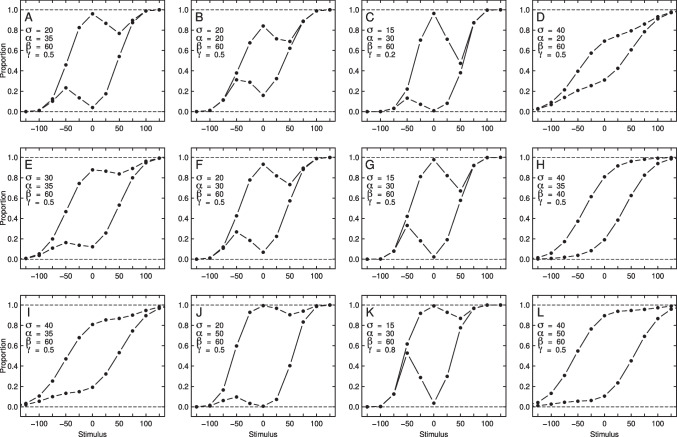
Three−Response Successiveness Model: Simulated Psychometric Functions

## Data Availability

The data used in this study are contained in Tables S2, S3, and S6 of the Supplementary Material for Kelber & Ulrich (2024), at 10.3758/s13414-024-02915-5.

## Appendix A

R Script for Estimation of PMF Cumulants

## Appendix B: R Scripts for Simulation of Three Models

Here we provide R scripts that produce simulated psychometric functions for the Four- Response Successiveness Model, the Threshold Model, and the Three-Response Successiveness Model.

Simulation of the Four-Response Successiveness Model

Simulation of the Threshold Model

Replace the select.response function of the Four-Response Successiveness Model with:

Simulation of the Three-Response Successiveness Model

Replace the select.response function of the Four-Response Successiveness Model with:

## Appendix C: Psychometric Functions from Three Models

Figures C1, C2, and C3 provide examples of psychometric functions from the Threshold Model, the Four-Response Successiveness Model, and the Three-Response Successiveness Model, respectively. The parameter values used to simulate the PMFs are shown on each plot. Where feasible, each column of three plots shows the effect of changing just one of the parameters.

## Appendix D: Three-Response Successiveness Model: Cumulants with a General Guessing Parameter (0 ≤ *γ* ≤ 1)

Table D.1

**Table D.1:** Three-Response Successiveness Model. *D*_*i*_ Cumulants [*κ*_*ij*_ = *κ*_*i*_(*D*_*j*_)]

Cumulant	Distribution	
	*D*_1_	*D*_2_
*κ*_1_	*β*(1 − 2*γ*) − 2*α* (1 − *γ*)	*β* (1 − 2*γ*) + 2* αγ*
*κ*_2_	*β*^2^ − ${\kappa }_{11}^{2}$	*β*^2^ − ${\kappa }_{12}^{2}$
*κ*_3_	(1 − 2*γ*)*β*^3^ − 3*β*^2^* κ*_11_ + 2${\kappa }_{11}^{3}$− − 2* α*^3^(1− γ)	(1 − 2*γ*)*β*^3^ − 3*β*^2^* κ*_12_ + 2${\kappa }_{12}^{3}+2\alpha$^3^*γ*

## Appendix E: Additional Tests of the Three-Response Successiveness Model

To use Eq. (11) to provide additional tests of the Three-Response Successiveness Model, applied to data from the van Eijk et al. (2008) study, it was necessary to relax the unbiased guessing (γ = 0.5) assumption, because Kelber & Ulrich (2024) had found that estimates of the guessing parameter (as well as *α* and *β*) varied considerably across subjects. We applied the tests to data from the selected eight subjects (Section 11), using the individual subject parameters provided by Paul Kelber and Rolf Ulrich, with the expressions in Appendix D used for the **D**_*i*_ cumulants. Values of the fourth cumulants, for which we do not have algebraic expressions, were computed numerically. We evaluated the extent to which the Eq. (11) contrasts depart from zero for each of the eight subjects separately, permitting the assessment of between-subject variability, with the results shown in Table E.1, which includes p-values from t-tests with 7 df.

**Table E.1:** *κ*_*m*_-*contrasts:* [*κ*_*m*_(**F**_1_) − *κ*_*m*_(**D**_1_)] − [*κ*_*m*_(**F**_2_) − *κ*_*m*_(**D**_2_)], *m* = 1, 2, 3, 4.

Contrast	Mean	SE	*p*-value
*κ*_1_ − *contrast*	1.47	1.12	0.23
*κ*_2_ − *contrast*/10^3^	−0.39	0.78	0.63
*κ*_3_ − *contrast*/10^5^	8.74	4.65	0.10
*κ*_4_ − *contrast*/10^8^	0.50	1.18	0.69

These results show that the test is satisfied by all four contrasts, as none of them differs significantly from zero.

## Footnotes

1 If the shape is known (or assumed), the spread can be estimated.

2 There is controversy as to whether multiple criteria on a perceptual dimension, required for multiple ratings, are less stable than a single criterion, resulting in a loss of sensitivity. Comparing ROC curves generated from binary decisions versus ratings, Egan, Schulman, & Greenberg (1959) showed no sensitivity loss in an auditory experiment, while Swets, Tanner, and Birdsall (1961) showed loss in a visual experiment. In their review, Green and Swets (1966, Sections 4.5, 11.2) conclude that there is minimal loss of sensitivity.

3 One way to think about judging the order of two events reported as simultaneous is as a response to the question “If you had to guess, which do you think came first?”

4 Cumulants, *κ*_*m*_, can be expressed as functions of moments, with *κ*_1_ = µ_1_′, *κ*_2_ = µ_2_, *κ*_3_ = µ_3_, and *κ*_4_ = µ_4_ − $3{\mu }_{2}^{2}$, where µ_1_′ is the mean, and the {µ_*m*_}, *m* ≥ 2, are moments about the mean. We refer interchangeably to the cumulants of a random variable, such as *κ*_*m*_(**F**_1_), and to those of its distribution function, i.e., *κ*_*m*_(*F*_1_).

5 The “arrival times” of two stimuli are the pair of times associated with them that enter into the judgment of their order, or, on some accounts, the times at which their representations arrive at the place in the brain where their order is judged.

6 Actually, there is no need in this generalization for *D* to be a nondecreasing function. (It would seem that few plausible models would violate this condition; but see Sections 8 and 9 for two such models). If *D* rises from zero to one nonmonotonically, it cannot be regarded as a distribution function of an actual random variable. Nevertheless, *F* is given by the convolution of *D* with the distribution function of **S** = **A**_*y*_ − **A**_*x*_, and the calculation used to determine the cumulants of **D** still produces quantities that contribute additively to the corresponding cumulants of **F**. (Under these conditions, F may or may not be monotonic, depending on details of **S** and **D**). Because such a **D** contributes in the same way to **F** as an actual random variable, it can be described as a “virtual random variable”.

7 If it is believed or assumed that the distribution of **S** is approximately Gaussian, supported for temporal-order judgments by Kelber & Ulrich (2024), then it is worth noting that for the Gaussian distribution, *κ*_3_ = *κ*_4_ = 0, so that *κ*_3_ and *κ*_4_ of the expected PMFs will be just those of the decision process.

8 For six such models applied to temporal-order perception, see Section II.C of Sternberg & Knoll (1973).

9 An application of these tests to the Three-Response Successiveness Model is provided in Appendix E.

10 Distribution functions are usually monotonic, whereas PMFs may not be. The computations go through nonetheless.

11 From its first four cumulants, the **S** distribution itself can be estimated, using Fleishman’s (1978) power method. See also Helwig (2022).

12 Again, as discussed in footnotes 6 and 10, if *D*_*i*_ is nonmonotonic, it must be regarded as a distribution function of a “virtual” rather than “actual” random variable, but the arguments go through in the same way.

13 Here and elsewhere in this paper, “**≈**” means “has the same distribution as”.

14 For certain “pathological” distributions of **L**_*y*_ − **L**_*x*_, parallel *F*_*i*_ may not require parallel *D*_*i*_. Strictly speaking, then, such distributions must be excluded. This can be done by adding the condition that **L**_*y*_ − **L**_*x*_ have a finite mean – a condition that will be satisfied in all cases of interest.

15 This model differs from the general threshold model considered by Ulrich (1987), in which the threshold can fluctuate and need not be centered around **S** = **A**_*y*_ − **A**_*x*_ = 0.

16 Because µ_2_(*F*_2_) is greater than µ_2_(*F*_1_) and µ_2_(*F*_3_), the standardized third cumulant, ${\mu }_{3}/{\mu }_{2}^{1.5}$, a measure of skewness, will be smaller for *F*_2_ than for *F*_1_ or *F*_3_.

17 In applying this model to perception of a different stimulus feature, such as pitch, perception of successiveness in the model would correspond to perceiving a difference between the two pitches, and perceiving order in the model would correspond to the discrimination the direction of the difference, i.e., which pitch is higher.

18 The relation between order and successiveness perception is controversial. See, e.g., Allan (1975a), Arstila, et al. (2020), Garcia-Perez & Alcala-Quintana (2012), Hirsh (1959), Love, et al. (2013), Machulla, et al. (2016), and Miyazaki, et al. (2016).

19 See footnote 3.

20 See footnotes 10 and 12.

21 As mentioned in Section 1, these variance and skewness features characterize the *F*_*i*_ found in Allan’s two studies.

22 The simulation programs would have to be slightly modified to permit variation of the Gaussian parameters, and more seriously modified if an alternative to the Gaussian distribution of sensory values was considered.

23 If a PMF fails to cover the full range of proportions from 0.0 to 1.0, one explanation is that the range of *d*-values was too small; in a better experiment, a sufficiently large range of *d*-values would be used to avoid this problem. A plausible alternative reason is that the observer was inattentive on some trials, and made a response – a “guess” – that was independent of the stimulus, except perhaps when the discrimination was especially easy. (It seems possible that if attention is “elsewhere”, and is returned to the task on presentation of the stimuli to be judged, but with a delay, the percept, degraded by the delay, may be useful for an easy discrimination, but not for a difficult one.) Such lapses of attention cause estimation difficulty even when the form of the PMF is known and only a threshold needs to be estimated (Green, 1995). Here the form is unknown and is the object of study, making it more important for experiments to use suitably timed warnings, performance-based payoffs, adequate practice, and other methods to minimize their occurrence.

24 Because the successiveness models can produce non-monotonic PMFs, cumulant estimation cannot use the modified Spearman-Kärber method (Sternberg, Knoll, & Zukofsky, 1982, Miller & Ulrich, 2001), which requires monotonicity.

## Code availability

Appendix A and C of the manuscript provide the relevant software.
